# Effect of Hydrogen on the Structure and Mechanical Properties of 316L Steel and Inconel 718 Alloy Processed by Selective Laser Melting

**DOI:** 10.3390/ma15144806

**Published:** 2022-07-09

**Authors:** Igor P. Maksimkin, Arkadiy A. Yukhimchuk, Igor L. Malkov, Igor E. Boitsov, Rafael K. Musyaev, Aleksey V. Buchirin, Victor V. Baluev, Anton V. Vertei, Evgeniy V. Shevnin, Sergey V. Shotin, Vladimir N. Chuvil’deev, Mikhail Yu. Gryaznov

**Affiliations:** 1 RFNC-VNIIEF, Nizhny Novgorod Region, 607190 Sarov, Russia; igor_m_13@mail.ru (I.P.M.); yukhim_ark@mail.ru (A.A.Y.); malk1951@mail.ru (I.L.M.); boitsov63@mail.ru (I.E.B.); rmusyaev@rambler.ru (R.K.M.); welr@mail.ru (A.V.B.); arkad@triton.vniief.ru (V.V.B.); antony48@yandex.ru (A.V.V.); evshevnin@vniief.ru (E.V.S.); 2 Materials Science Department, Physical and Technical Research Institute, Lobachevsky State University of Nizhny Novgorod, 603105 Nizhny Novgorod, Russia; chuvildeev@nifti.unn.ru (V.N.C.); gryaznov@nifti.unn.ru (M.Y.G.)

**Keywords:** selective laser melting, hydrogen permeability, mechanical properties, Inconel 718, 316L stainless steel

## Abstract

The interaction of hydrogen with specimens of 316L steel and Inconel 718 alloy processed by selective laser melting (SLM) was studied. The effect of hydrogen on the mechanical properties of SLM materials, hydrogen permeability, and microstructure was investigated; besides, these values were compared with the properties of conventionally produced materials. It was shown that SLM can be successfully used to produce parts for operation in hydrogen environments at high pressure at room temperature.

## 1. Introduction

Currently, materials produced by selective laser melting (SLM) technologies are regarded as a new class of structural materials, while SLM is considered as an advantageous technology for designing complex-shaped metal products for prospective applications in mechanical engineering, biomedicine, the power industry, etc. [1,2,3,4,5]. Active research is underway into SLM materials and by now the effect of melting modes on the density, structure, and physical and mechanical properties of various SLM materials has been studied comprehensively [6,7,8,9,10]. Their corrosion resistance [2,4,9,10,11], deformation behavior at elevated temperatures [5,8,12,13,14], resistance to fatigue failure [3,8,11,15,16,17], and other properties are being actively investigated.

At present, 316L stainless steel and Inconel 718 nickel superalloy are the most commonly used materials in additive manufacturing. The mechanical properties of SLM-processed 316L steel and Inconel 718 alloy have been extensively researched [6,8,14,18,19]. Studying the interaction of such materials with hydrogen is a new attractive research field [20,21,22]. The results of prospective studies are of interest for manufacturing equipment used in hydrogen-containing environments, in particular, high-pressure vessels.

The current paper aimed to study the interaction of SLM-processed 316L stainless steel and Inconel 718 nickel alloy with hydrogen as well as to study the hydrogen effect on mechanical properties and hydrogen permeability.

## 2. Materials and Methods

The objects of this research were specimens of 316L corrosion-resistant austenitic steel and Inconel 718 nickel superalloy processed by selective laser melting of powder materials. Table 1 shows the chemical composition of the powders.

**Figure 1:**
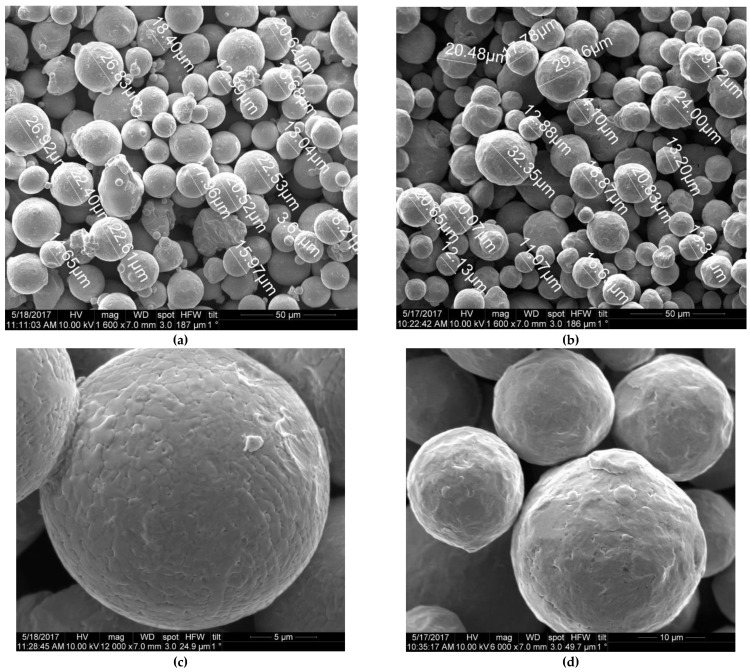
SEM image of Inconel 718 alloy (**a**,**c**) and 316L austenitic steel (**b**,**d**) powders at different magnifications.

The SALD-2300 Shimadzu laser particle-size analyzer was used to qualify particle size distribution of the powders. The average particle size of 316L and Inconel 718 powders was 15 and 20 µm respectively (Figure 1). The D_75_ values for 316L steel and Inconel 718 alloy powders were under 20 and 25 µm respectively (75% of the powder particles had a diameter of less than 20 and 25 µm).

**Figure 2:**
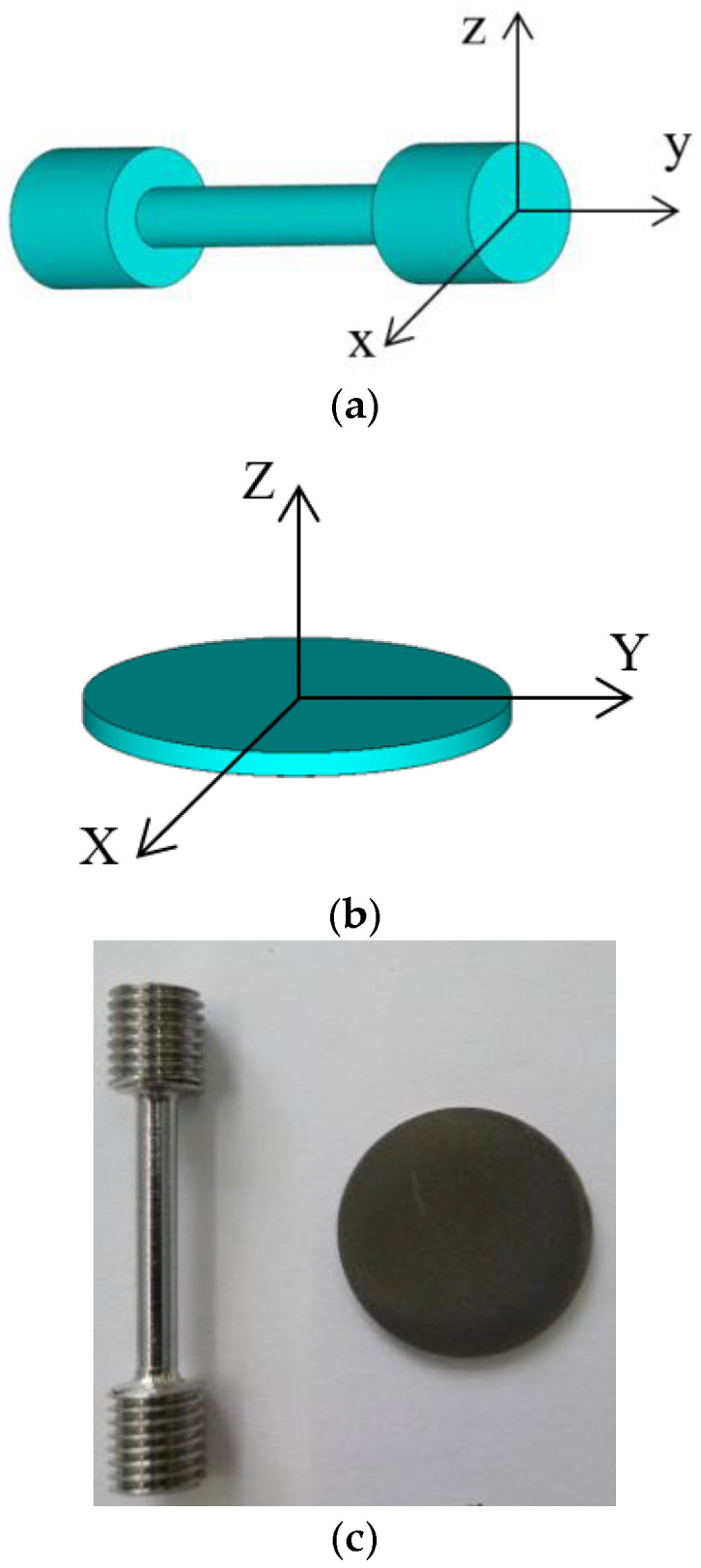
SLM specimen image: (**a**)—tensile test specimens, (**b**)—hydrogen permeability test specimens, (**c**)—photos of (**a**) and (**b**) specimens.

Specimens were processed using the Realizer SLM 100 machine. The basic parameters of the SLM process are shown in Table 2. Two types of specimens were processed for the research: cylindrical specimens for tensile tests (Figure 2a) and membrane specimens for studying hydrogen permeability (Figure 2b). During manufacturing, the axis of cylindrical specimens for tensile testing was perpendicular to the laser beam axis, while that of disk-shaped specimens for hydrogen permeability studies was parallel to the laser beam axis. Tensile tests were performed on Type III cylindrical specimens with a diameter and length of the gauge section of 3 and 15 mm respectively (ISO 6892-1). Dog-bone shaped specimens for tensile tests were produced by turning the SLM billets (Figure 2c). One-millimeter thick membrane specimens with a diameter of 20 mm for studying hydrogen permeability were produced by wire electrical discharge machining from cylindrical billets 20 mm in diameter and 10 mm in length. There was no additional surface treatment of the membrane specimens.

The microstructure of the specimens was studied with the Axiovert 25 optical microscope after etching in a hot Krupp’s reagent (50 mL of hydrochloric acid, 5 mL of nitric acid, and 5 mL of water). The microstructure of cylindrical specimens was studied in three planes XZ, YZ, and XY (see Figure 2a), while the microstructure of membrane specimens was researched in sectional planes XZ and XY (Figure 2b).

The mechanical properties of specimens and the effect of high-pressure hydrogen were determined during tensile tests in helium and in hydrogen (with purity of ≥99.9999%) at 80 MPa pressure and room temperature. The specimens were kept in hydrogen for 10 min before testing. The UTS 100 K testing machine, equipped with a chamber for testing in high-pressure gas environments, was used to perform tensile tests at a strain rate of 5 × 10^−5^ s^−1^. The chamber, the testing techniques, and measurement errors are described in refs. [23,24]. The following mechanical properties were determined during the tests: σ_B_—ultimate tensile strength, σ_0.2_—yield stress, δ_5_—elongation to failure, ψ—percentage reduction of area. The relative error of measuring σ_B_ and σ_0.2_ was 4.7%, with the absolute error of measuring δ_5_ and ψ being 0.1% and 0.4% respectively. The hydrogen effect on mechanical properties was evaluated through a non-dimensional parameter: β = X (H2)/X (He), where X (H2) is the value of the (σ_B_, σ_0.2_, δ_5_, ψ) characteristic during tensile tests in hydrogen and X (He) is its value during tests in helium.

**Figure 3:**
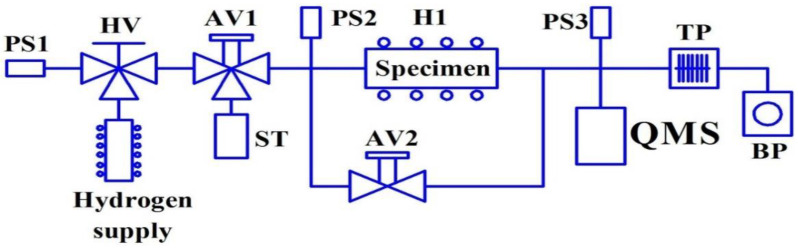
Permeability testing system: PS1, PS2, PS3—pressure sensors; HV—hand valve; AV1, AV2—air valve; H1—heater; ST—surge tank; QMS—mass spectrometer; TP—turbomolecular pump; BP—backing pump; hydrogen supply—vanadium metal hydride hydrogen generator.

**Figure 4:**
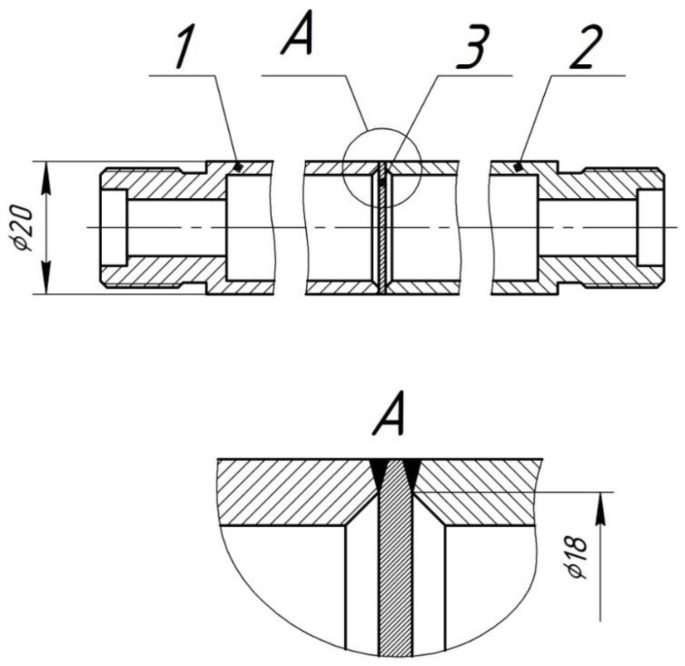
Model assembly for high-temperature hydrogen permeability tests: 1, 2—tubes; 3—researched specimen (membrane).

Hydrogen permeability of membrane specimens was studied on a special testing unit (Figure 3). The membrane specimen was laser-welded between two stainless steel tubes with a diameter of 20 mm and 2 mm-thick walls (effective specimen diameter ~18 mm) (Figure 4).

Before the high-temperature hydrogen permeability tests, the specimens were annealed in vacuum at constant pumping at a residual pressure less than 5 × 10^−3^ Pa and at a temperature of 100–150 K higher than the testing temperature (up to 1273 K during 4 min and up to 1023 K for 2.5 h). Once annealing was complete and the target temperature of the specimen was reached, diffusion-pure hydrogen was supplied to the input membrane surface of the specimen at a pressure of ~0.5 MPa for 316L steel and ~0.9 MPa for Inconel 718 alloy. The pressure value was set based on the strength properties of the membrane specimen material when used within the elastic region. The QMS Prisma 200M quadrupole mass spectrometer was used to record the flow (partial pressure) of hydrogen passing through the specimen at output membrane surface. The pressure at the input surface of the specimen remained constant throughout the experiment. Based on the analysis of the experimental data obtained by the method of determining high-temperature hydrogen permeability [25] (based on the Daynes method), the following kinetic parameters of hydrogen permeability were calculated: P_H_—hydrogen permeability coefficient; E_P_—hydrogen permeability activation energy; D_H_—diffusion coefficient; E_D_—diffusion activation energy; S_H_—hydrogen isotopes solubility in the material; H_S_—heat of dissolution.

X-ray diffraction analysis (XRD) was conducted using the Shimadzu LabX XRD-6000 diffractometer (radiation CuK_α_, λ = 1.54056 Å, the 2θ range from 20° to 80°, scanning speed 2°/min—for survey X-ray, 0.125°/min—for studying X-ray peak broadening).

## 3. Results

### 3.1. Results of Mechanical Tests

#### 3.1.1. Tensile Test Results for 316L Steel Specimens

**Figure 5:**
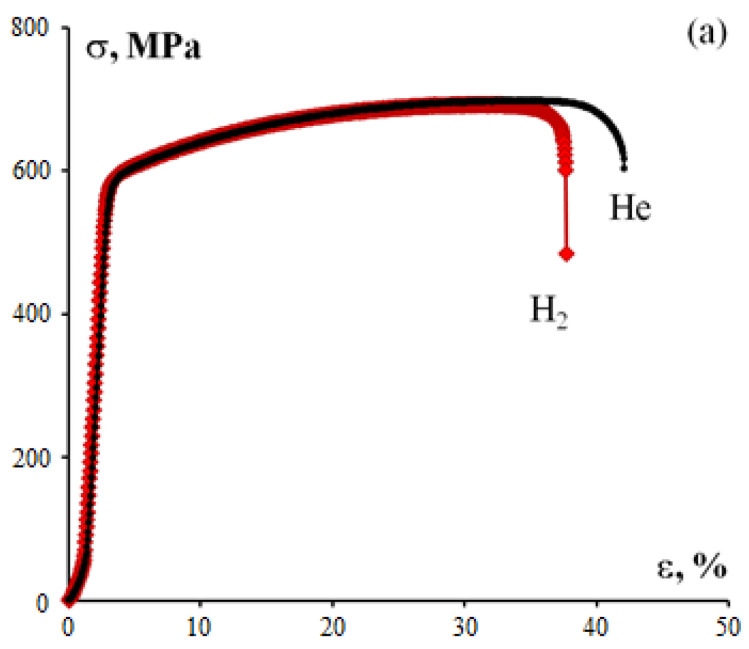

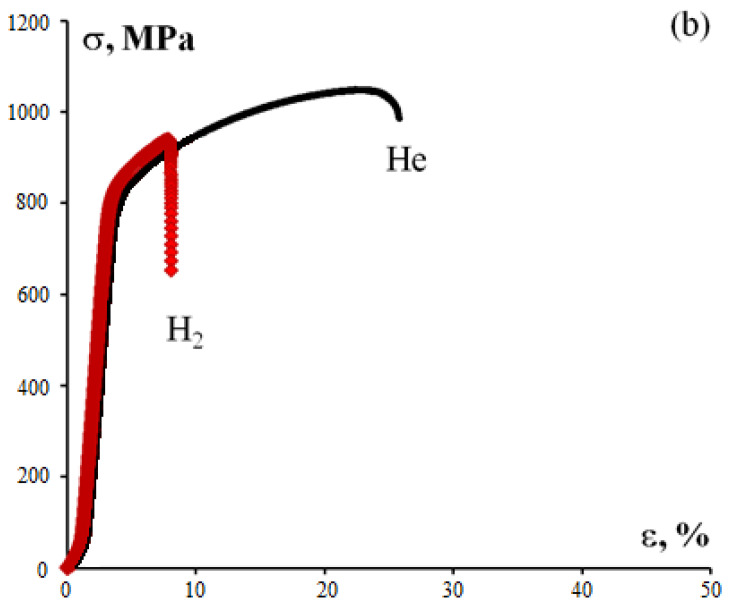
Stress–strain diagrams in helium and in hydrogen for 316L steel (**a**) and Inconel 718 alloy (**b**) specimens.

Table 3 shows the tensile tests results for 316L steel specimens in hydrogen and in helium at 80 MPa. Figure 5a shows tensile stress–strain diagrams (σ–ε) for 316L steel specimens in helium and in hydrogen. As is demonstrated in Figure 5a, σ (ε) relations for SLM 316L steel are characterized by a long stage of stable plastic flow. It should be noted that for testing in hydrogen, the σ (ε) relation remains unchanged with a slight decrease in plasticity.

For comparison, Table 3 shows the results of tensile testing in helium and in hydrogen of specimens made of 1.4404 steel (analogous to 316L steel) obtained by hot rolling [23,24]. As can be seen from Table 3, SLM-processed 316L steel has high mechanical properties. It should be pointed out that SLM 316L steel exhibits higher strength (yield stress, in particular) and lower plasticity than wrought steel.

An analysis of the results in Table 3 suggests that SLM-processed 316L steel specimens have better hydrogen-resistance characteristics. Ultimate tensile strength, yield stress, and elongation to failure decrease by less than 5%–10% against the typical values. Percentage reduction of area is the most sensitive to hydrogen exposure. Despite a noticeable decrease during tests in hydrogen (22% of the typical value), percentage reduction of area remains very high (ψ = 46%). It should be also noted that plasticity reduced less in SLM-processed 316L steel than in similar steel manufactured conventionally.

#### 3.1.2. Tensile Test Results for Inconel 718 Alloy Specimens

Table 4 shows the tensile tests results for Inconel 718 specimens in hydrogen and in helium at 80 MPa. Figure 5b shows the tensile test diagrams of Inconel 718 alloy specimens in helium and in hydrogen. As is shown in Figure 5b, σ (ε) relations for SLM-processed Inconel 718 alloy are characterized by a long strain-hardening stage followed by fast fracture of the specimens. The type of σ (ε) relations during testing in helium and in hydrogen was similar whereas Inconel 718 alloy demonstrated lower plasticity when tested in hydrogen.

These results suggest that hydrogen does not significantly affect the strength characteristics of the Inconel 718 alloy. It should be noted that Inconel 718 is a precipitation-hardened and heat-hardened alloy, demonstrating σ_B_ ≥ 1250 MPa and σ_0.2_ ≥ 900 MPa after standard heat treatment [26]. SLM specimens were not subjected to additional hardening heat treatment, so their strength properties were 15% lower than for heat-hardened specimens.

It should be noted that during tests in hydrogen, the Inconel 718 alloy specimens began to fail in the area of transition from the gauge section to the gripping head. This result confirms that SLM-processed Inconel 718 alloy specimens with stress concentrators are highly sensitive to hydrogen. This particular type of specimen failure where the fracture zone was outside the gauge section of the specimen prevented us from correctly determining the plasticity behavior of SLM-processed Inconel 718 alloy specimens and, as a result, did not allow us to evaluate the hydrogen effect on the plasticity of Inconel 718 alloy. It should be noted that some research (for example, ref. [27]) illustrates a significant effect of stress concentrators on mechanical properties of the wrought Inconel 718 alloy when tested in hydrogen-containing environments.

### 3.2. Results of Hydrogen Permeability Studies

**Figure 6:**
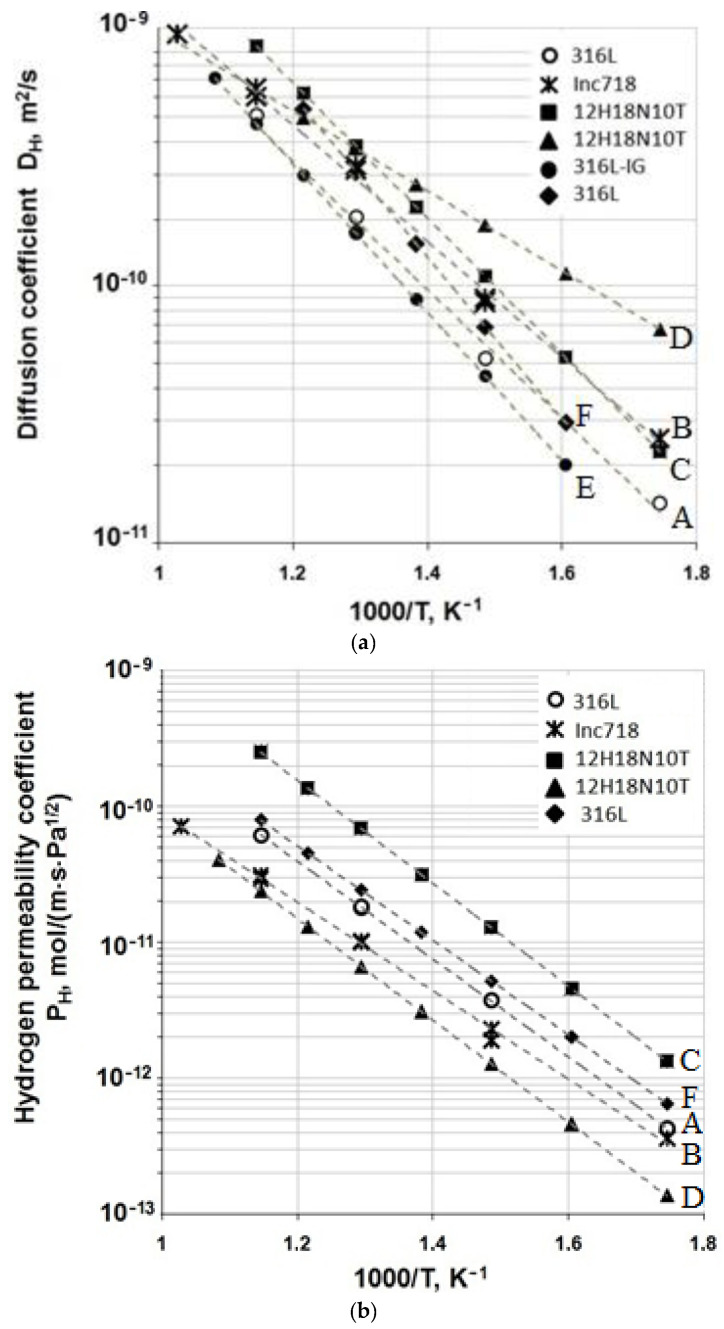
Temperature dependence of the diffusion coefficient (**a**) and permeability polytherm (**b**) for the researched materials. A,B—data for SLM-processed 316L and Inconel 718, respectivetly, obtained in this work, C—values for 12H18N10T from ref. [32], D—values for 12H18N10T from ref. [33], E—values for 316L-IG from ref. [34], F—values for 316L from ref. [35].

Table 5 contains hydrogen permeability kinetic parameters for SLM materials and literature data for similar conventionally produced materials. As an example, Figure 6 provides temperature dependences of hydrogen permeability coefficient P_H_ and diffusion coefficient D_H_ for 316L steel specimens.

**Table 5:** Kinetic parameters of high-temperature hydrogen permeability for SLM specimens and specimens processed conventionally.

Material	D_0_, m^2^/s	E_D_, kJ/mol	P_0_, mol/ (m⋅s⋅Pa^1/2^)	E_P_, kJ/mol	S_0_, mol/ (m^3^⋅Pa^1/2^)	H_S_, kJ/mol
316L steel	3.42 × 10^−7^	48.0	7.8 × 10^−7^	68.66	2.28	20.64
Inconel 718	1.66 × 10^−7^	41.9	1.4 × 10^−7^	61.33	0.84	19.47
12X18H10T steel [28]	8.60 × 10^−7^	50.2	5.48 × 10^−6^	72.5	-	15.90
12X18H10T steel [29]	3.40 × 10^−8^	29.6	4.5 × 10^−7^	71.4	-	-
316L-IG steel [30] (for deuterium)	5.90 × 10^−7^	55.0	0.3 × 10^−6^	66.0	0.50	11.0
316L steel [31]	2.99 × 10^−6^	59.7	7.7 × 10^−7^	66.6	0.26	6.88

An analysis of these results suggests that the kinetic parameters of hydrogen diffusion and permeability for SLM-processed 316L steel and Inconel 718 alloy are similar to those of 12X18H10T and 316L wrought steels. Somewhat low values of diffusion activation energy and high values of the heat of dissolution as compared with the data for conventionally produced materials can be possibly due to microscopic pores with passivated open surfaces in the structure formed during the SLM process.

### 3.3. Results of Metallographic Studies

**Figure 7:**
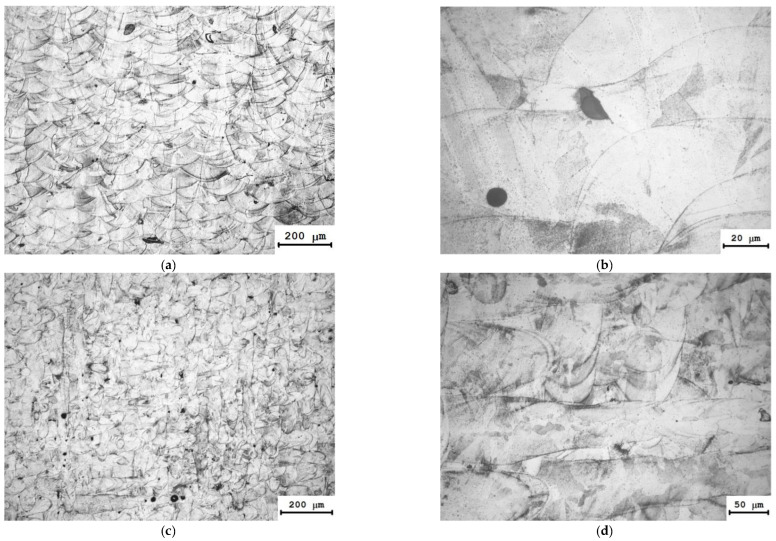
Microstructure of a steel 316L cylindrical specimen: (**a**,**b**)—XZ plane; (**c**,**d**)—XY plane.

The microstructure of 316L steel at magnification ×400 is a combination of crossing “microwelded seams” (i.e., laser beam scanning track), and it can be clearly seen using optical microscopy (Figure 7). Such microstructures are quite common in SLM-processed austenitic steels [36,37,38,39]. It should be noted that in contrast to the welded seam of 12X18H10T steel that has a dendritic structure formed during relatively slow cooling of the molten metal, dendrites are not observed in SLM 316L steel specimens. We expect that this may be due to the high solidification rate of the material; in ref. [32], the steel cooling rate after laser exposure was estimated at 10^3^ … 10^6^ K/s, which is much higher compared with the steel melt solidification rate for conventional casting methods. In our work, the characteristic size of microstructural elements (the width of microwelded seams) was about 100 µm (Figure 7).

The structure of 316L steel in the laser beam scanning plane (XY plane) (Figure 7c,d) is different from the structure of the material in the XZ and YZ planes that are parallel to the laser beam. Elliptical shaped areas were observed in the XY plane structure and their boundaries are clearly defined in X or Y scanning directions. These areas are the longitudinal section of the microwelded seams formed along the laser beam scanning directions. The 316L steel structure has single pores and lacks of fusion that do not significantly affect the mechanical properties of the steel specimens (see Table 3).

**Figure 8:**
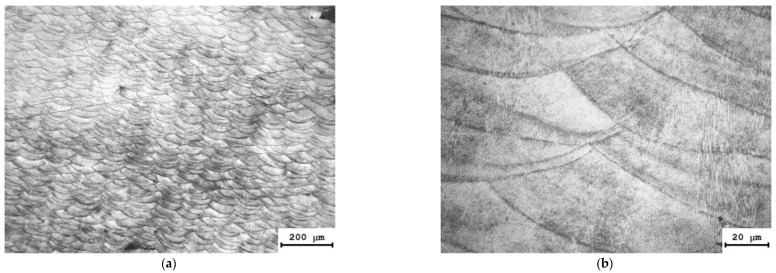
Microstructure of an Inconel 718 alloy cylindrical specimen (YZ plane) at different magnifications: (**a**) ×50: (**b**) ×500.

The mesostructure of the Inconel 718 alloy specimen consisting of crossing microwelded seams are similar to those of the 316L steel specimen (Figure 8), and the same structure of SLM Inconel 718 alloy is observed in refs. [33,34,35].

**Figure 9:**
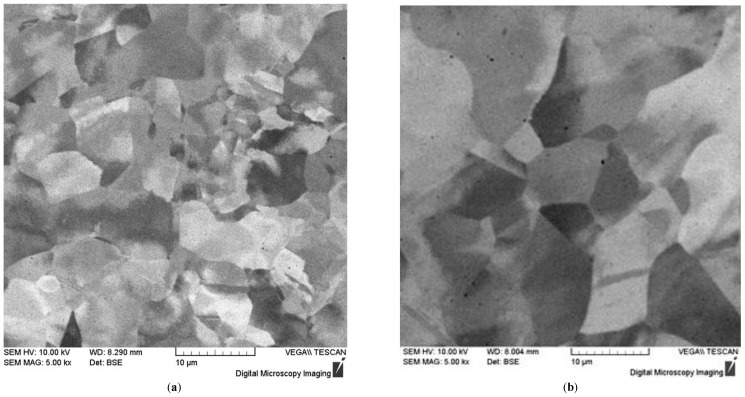

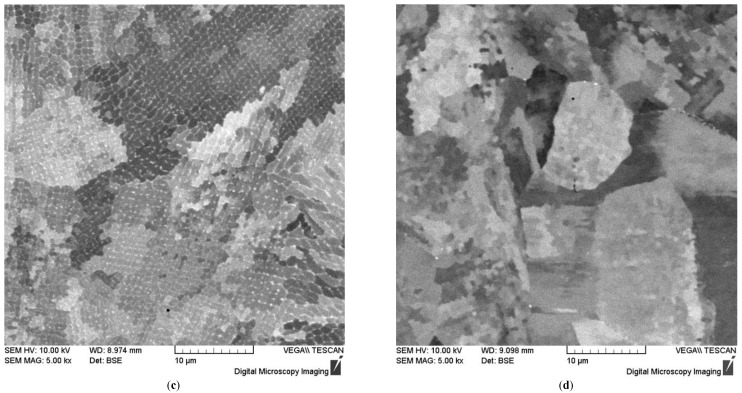
SEM images of the microstructure of SLM specimens of 316L steel (**a**,**b**) and Inconel 718 alloy (**c**,**d**) obtained before (**a**,**c**) and after (**b**,**d**) high-temperature hydrogen permeability tests (XY plane).

Figure 9a,b show the characteristic microstructures obtained on the 316L steel specimens before and after the high temperature hydrogen permeability tests. The average grain size was about 8 μm before the tests (Figure 9a). The microstructure of 316L steel was practically unchanged after high-temperature hydrogen permeability tests, a slight grain growth to an average size of 11 μm being observed (Figure 9b).

Figure 9c,d show the characteristic microstructure obtained on SLM Inconel 718. On the specimens before the high temperature hydrogen permeability tests (Figure 9c), a grain structure with an average grain size of about 10 μm and a cellular structure with an average cell size of about 2 μm were observed. The presence of cells in the Inconel 718 alloy is usually associated with the Laves phase [40,41]. The grain structure of Inconel 718 alloy did not change significantly after high-temperature hydrogen permeability tests, a slight increase in the average grain size up to 15 μm being observed (Figure 9d). It should be noted that the cells of the Laves phase disappeared completely after the high-temperature hydrogen permeability tests.

Changing grain structure in SLM materials after high-temperature hydrogen permeability testing indicates the start of a recrystallization process. Recrystallization normally occurs after heating the deformed material due to a decrease in the elastic strain energy [42,43]. In SLM-processed specimens, rapid solidification may cause residual stresses [44,45,46,47,48], which create a certain reserve of elastic energy. When a specimen is heated to high temperatures (prior to hydrogen permeability testing, the membrane specimen was subjected to high-temperature treatment, which was a guarantee of reliable degassing of the membrane and chamber walls from residual hydrogen), recrystallization processes of the material may be activated. Changes in the membrane specimen structure as a result of high-temperature hydrogen permeability testing are accompanied by a decrease in the microhardness of 316L steel from 2600 to 2100 MPa, which also confirms the beginning of recrystallization. It should be noted, however, that the microhardness of steel after high-temperature hydrogen permeability testing remains very high compared with the microhardness of the base metal and weld joints of 316L steel specimens [49].

**Figure 10:**
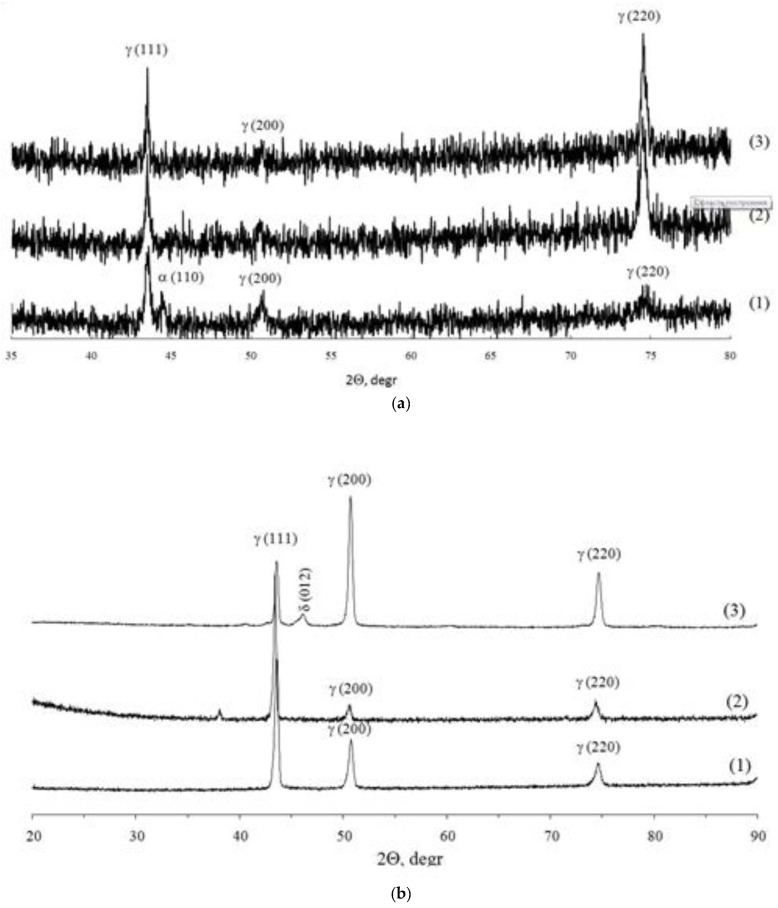
X-ray phase analysis results of 316L steel (**a**) and Inconel 718 alloy (**b**) specimens in various structural states: (1) powder; (2) SLM specimen; (3) SLM specimen after high-temperature testing in hydrogen.

The recrystallization process during high temperature tests of 316L and Inconel 718 alloy specimens in a hydrogen environment was confirmed by the X-ray diffraction analysis results. Figure 10a shows X-ray diffraction patterns for 316L steel in various states (powder, SLM specimen, and SLM specimen after high-temperature tests in a hydrogen environment), and Figure 10b demonstrates the results of X-ray diffraction analysis of Inconel 718 alloy specimens. (The high intensity of -Fe austenite X-ray peak (220) at large diffraction angles is linked to the texture of the specimen. Hence, the effect of internal stresses on the X-ray peak broadening in the area of large diffraction angles was not performed).

An analysis of the data in Figure 10a suggests that after SLM, the half-width of the (111) peak of γ-Fe austenite in SLM 316L steel decreased from 0.286° to 0.241° and it was further reduced to 0.236° after high-temperature testing in hydrogen. This result indicated a decrease in internal microstresses of the second order during SLM and high-temperature annealing. It should be noted that the position of the (111) peak in this case changed insignificantly—the diffraction angle 2Θ_max_, corresponding to the position of the (111) γ-Fe austenite maximum X-ray peak in 316L steel for the powder and SLM specimen was 43.42° (±0.02°), with a slight increase in 2Θ_max_ to 43.46° (±0.01°) after high-temperature tests in hydrogen. The shift of the X-ray maximum towards large reflection angles due to the Wulff–Bragg’s equation (2d⋅sin(Θ_max_) = nλ, where d is interlayer spacing, λ is the X-ray wavelength, and *n* is the X-ray diffraction maximum number), suggests that after SLM-compressive internal macrostresses (stresses of the first order) were formed in the 316L steel specimen, they favorably affected the mechanical properties of SLM parts.

Similar results were obtained from X-ray diffraction analysis of Inconel 718 alloy specimens (Figure 10b)—2Θ_max_ in Inconel 718 alloy after SLM increased from 43.40° (±0.01°) to 43.44° (±0.01°) with an increase of 2Θ_max_ = 43.50° (±0.02°) after a high-temperature test in hydrogen. The half-width of the X-ray peak (110) then decreased from 0.191° (powder of Inconel 718) to 0.140° (SLM specimen after high-temperature testing in hydrogen).

## 4. Conclusions

1. At room temperature in a hydrogen environment, the mechanical properties of 316L steel processed by SLM exceed those of steel produced conventionally. In particular, the ultimate tensile strength and yield stress were 690 and 570 MPa respectively. The hydrogen effect leads to a slight decrease in the strength of SLM 316L steel by 5%, which is comparable to a strength decrease in similar steels manufactured using conventional technology.
2. Hydrogen exposure does not have a significant effect on the strength of SLM-processed Inconel 718 alloy. However, the fracture behavior of SLM specimens shows the high sensitivity to stress concentrators in hydrogen. Ultimate tensile strength and yield stress for SLM Inconel 718 (as-built) were 940 and 790 MPa respectively in a hydrogen environment.
3. The kinetic parameters of hydrogen permeability for SLM 316L steel and Inconel 718 alloy specimens were comparable to those of 12X18H10T and 316L steel specimens produced conventionally. Small differences in the activation energies of diffusion and hydrogen dissolution, caused by the SLM process, did not significantly affect the processes of hydrogen transfer in the researched materials.
4. The obtained results confirm that SLM can be successfully used to produce parts for operation in hydrogen environments at high pressure at room temperature.

## Figures and Tables

**Table 1:** Chemical composition of 316L steel and Inconel 718 powders (wt. %).

Material	Fe	Ni	Cr	Mo	Nb	Mn	Ti	Al	Si	C	S	P
316L steel	Bal.	14	17.0	2.8	-	1.5	0.25	-	0.40	0.03	0.01	0.02
Inconel 718	19	Bal.	19.9	4.1	5.3	0.3	1.0	0.8	0.25	0.04	0.01	0.01

**Table 2:** Basic parameters of selective laser melting.

Parameter	316L	Inconel 718
Layer thickness, μm	30	30
Laser power, W	80	100
Exposure time, µs	80	100
Point distance, µm	50	30
Laser beam diameter, µm	90	100
Hatch spacing, µm	120	150
Angle rotation of scanning direction	90°	90°
Substrate preheating temperature	200 °C	
Protective environment	Argon (99.998 wt. %)	

**Table 3:** Tensile tests results for SLM 316L steel in hydrogen and in helium.

Material	Environment	σ_B_	σ_0.2_	δ_5_	ψ
		MPa		%	
316L steel (this work)	He (80 MPa)	730	600	41	59
	H_2_ (80 MPa)	690	570	37	46
	Parameter β	0.95	0.95	0.90	0.78
Tensile test results for similar steel specimens processed by conventional manufacturing methods [23,24]					
1.4404 steel (Ø 12 mm bar)	He (70 MPa)	590	265	63	85
	H_2_ (70 MPa)	570	260	45	43
	Parameter β	0.97	0.98	0.71	0.51
1.4404 steel (12 mm sheet)	He (70 MPa)	650	375	54	83
	H_2_ (70 MPa)	620	345	41	46
	Parameter β	0.95	0.92	0.76	0.55

**Table 4:** Tensile tests results for SLM Inconel 718 in hydrogen and helium at 80 MPa.

Environment	σ_B_	σ_0.2_	δ	ψ
	MPa		%	
He	1040	780	19.5	29
H_2_	940	790	-	-
Parameter β	0.90	1.01	-	-

## Data Availability

Not applicable.
